# Machine learning combined with resting-state functional MRI to characterize functional brain differences in post-stroke depression

**DOI:** 10.3389/fpsyt.2026.1835586

**Published:** 2026-06-22

**Authors:** Yuanxin Shao, Chao Liang, Dan Xu, Yang Zhao, Phi Thi Thanh Hoa, Xue Zhang, Dongyang Shi, Weifeng Guo

**Affiliations:** 1 First Clinical Medical College, Nanjing University of Chinese Medicine, Nanjing, Jiangsu, China; 2 Liaocheng People’s Hospital, Liaocheng, Shandong, China; 3 Taicang TCM Hospital Affiliated to Nanjing University of Chinese Medicine, Taicang, Jiangsu, China; 4 Dalian Medical University, Dalian, China; 5 Vietnam National Hospital of Acupuncture, Hanoi, Vietnam

**Keywords:** anxiety, depression severity, functional connectivity, machine learning, post-stroke depression, resting-state functional magnetic resonance imaging

## Abstract

**Background:**

Post-stroke depression (PSD) is a common neuropsychiatric condition after stroke, but its resting-state functional imaging correlates remain incompletely characterized. This study examined multi-level resting-state functional differences between patients with PSD and healthy controls and evaluated whether interpretable machine learning could identify candidate imaging features associated with PSD.

**Methods:**

Fifty patients with PSD and 50 age- and sex-matched healthy controls underwent resting-state functional MRI. Four complementary imaging indices were extracted using the AAL atlas: amplitude of low-frequency fluctuation (ALFF), regional homogeneity (ReHo), degree centrality (DC), and ROI-to-ROI functional connectivity (FC). Imaging features were adjusted for demographic variables, head motion, medication use, and stroke-related factors where appropriate. Candidate features with significant between-group differences were further reduced using LASSO regression. Nine machine-learning classifiers were trained and compared under the same feature-selection framework. Model performance was assessed primarily by ROC-AUC. SHapley Additive exPlanations (SHAP) were used to examine the contribution of individual features to the best-performing model.

**Results:**

Patients with PSD showed distributed resting-state functional differences involving cingulate, thalamic, prefrontal, insular, posterior default-mode, and visual-associated regions. Twenty-nine candidate features differed between groups, including 7 ReHo, 8 ALFF, 6 DC, and 8 FC features. LASSO retained 10 core features, with a cross-validated AUC of 0.878. Among the nine classifiers, the Extra Trees model achieved the highest independent test-set performance, with an AUC of 0.889. SHAP analysis indicated that the most influential features included DC in the left anterior cingulate and paracingulate gyri, ReHo in the left thalamus, FC between the left precuneus and left calcarine cortex, ALFF in the right precuneus, and ALFF in the left angular gyrus. Within the PSD group, moderate depression was associated with higher ALFF in the left insula and lower precuneus-calcarine connectivity compared with mild depression.

**Conclusions:**

Patients with PSD showed multi-level resting-state functional differences compared with healthy controls. Interpretable machine learning identified a set of candidate rs-fMRI features with plausible neurobiological relevance. These findings require validation in larger longitudinal cohorts that include post-stroke patients without depression to clarify their specificity and clinical utility.

## 1 Introduction

Stroke is one of the leading causes of death and long-term disability worldwide. Approximately 30%–50% of stroke survivors develop emotional and cognitive disturbances during recovery, which substantially impair functional outcomes and quality of life (1). Among these non-motor complications, post-stroke depression (PSD) is particularly common. It is regarded as one of the most important yet frequently overlooked neuropsychiatric syndromes after stroke. Epidemiological studies have shown that nearly one third of stroke survivors meet diagnostic criteria for depressive disorder at different stages after stroke. PSD is closely associated with poorer adherence to rehabilitation, worse functional recovery, and increased mortality risk. It also imposes a substantial medical and social burden, making it a major concern in neurology, psychiatry, and public health (2).

Current evidence generally suggests that PSD is not merely a consequence of psychological stress or reactive emotional disturbance. Rather, it arises from the combined effects of stroke-related structural injury, biological susceptibility, and functional network reorganization. With advances in neuroimaging, resting-state functional magnetic resonance imaging (rs-fMRI) has become an important tool for investigating depression and stroke-related psychiatric disorders because it does not require task performance, shows good reproducibility, and can characterize spontaneous whole-brain activity and functional network organization. A large body of evidence indicates that depressive disorders are closely related to abnormal interactions among the default mode network (DMN), salience network (SN), and executive control network. This model of network imbalance has become one of the central frameworks for understanding the neural basis of depression (3, 4). In the context of stroke, focal lesions may affect widespread functional connectivity and network topology through remote effects, thereby triggering or amplifying depression-related network abnormalities. This view has been increasingly supported by neuroimaging studies (5).

Previous rs-fMRI studies of PSD have mainly focused on a single metric or a single network level, such as local spontaneous activity, interregional functional connectivity, or connectivity changes within a specific network. These studies have provided important clues to the neural basis of PSD, but their findings are often heterogeneous, and some results are difficult to reproduce across samples or analytic strategies. On the one hand, a single metric cannot fully capture the multiscale neural alterations involved in PSD. On the other hand, stroke-induced network reorganization may simultaneously affect local activity, local synchrony, whole-brain hub properties, and interregional coupling. Analyses confined to one level may therefore underestimate the complexity of PSD-related dysfunction (6, 7). Accordingly, integrating multiple complementary rs-fMRI metrics to characterize brain functional abnormalities in PSD across different levels has become an important unresolved issue in this field.

In addition, although machine learning has been widely introduced into psychiatric neuroimaging for individual-level disease identification and risk prediction, two major challenges remain in PSD research. First, there is still no unified comparison across different algorithms and feature construction strategies, and model performance and stability vary substantially. Second, many studies emphasize classification accuracy while paying insufficient attention to model interpretability. As a result, imaging features are difficult to trace back to specific brain regions or functional networks, which limits their biological significance and clinical translational value (8, 9). In depression research, there is increasing consensus that interpretability is not optional. It is a critical bridge between algorithmic performance and mechanistic understanding, especially for disorders characterized by prominent network pathology (10).

Against this background, several important gaps remain in PSD research. First, there is still a lack of a systematic framework that can describe PSD-related functional abnormalities simultaneously across multiple levels, including local amplitude, local synchrony, whole-brain network hubs, and interregional coupling. Second, it remains unclear whether PSD-related functional alterations reflect coordinated imbalance across networks rather than isolated regional abnormalities. Third, how to translate multidimensional imaging phenotypes into stable and interpretable individual-level features remains a key challenge for the clinical application of PSD neuroimaging.

Therefore, the present study primarily aimed to clinically characterize whole-brain functional abnormalities in post-stroke depression by comprehensively comparing patients with PSD and healthy controls across multiple resting-state fMRI metrics, including ALFF, ReHo, DC, and FC. This design enabled a multidimensional assessment of PSD-related alterations in local brain activity, local synchrony, hub properties, and interregional functional coupling. As a secondary and exploratory objective, we incorporated interpretable machine learning to identify the most informative neuroimaging features for PSD classification and to improve the biological interpretability of model performance. Specifically, nine machine learning models were compared, and the best-performing model was further interpreted using SHAP to determine the brain regions and network features contributing most strongly to classification. In addition, subgroup analyses based on symptom severity were performed to further explore brain functional alterations associated with clinical heterogeneity in PSD. Through this framework, we aimed to provide a comprehensive clinical imaging description of PSD, complemented by exploratory identification of key imaging biomarkers and further characterization of severity-related functional differences.

## 2 Methods

### 2.1 Study design and participants

This was a single-center, prospective case-control study. A total of 50 patients with PSD were consecutively recruited from Liaocheng People’s Hospital between January 1, 2024 and December 12, 2025. In parallel, 50 healthy controls (HCs) were recruited using frequency matching for age and sex. The study was conducted in accordance with the Declaration of Helsinki. The protocol was approved by the Ethics Committee of our hospital (2023229), and all participants provided written informed consent before enrollment.

### 2.2 PSD group: inclusion and exclusion criteria

#### 2.2.1 Inclusion criteria

Participants were eligible for the PSD group if they met the following criteria: age 18–75 years; a diagnosis of stroke, defined as an acute focal or global neurological deficit of vascular origin, with ischemic or hemorrhagic lesions confirmed by CT or MRI (11); unilateral supratentorial stroke lesions; PSD diagnosis was made by two qualified psychiatrists based on clinical interview and symptom assessment, with a 17-item Hamilton Depression Rating Scale (HAMD-17) score of 8-24, indicating mild to moderate depressive symptoms (12); first-ever stroke; and a disease duration of 2 weeks to 1 year.

#### 2.2.2 Exclusion criteria

Participants were excluded if they had any of the following: a history of other major central nervous system diseases, such as epilepsy, brain tumor, multiple sclerosis, Parkinson’s disease, or severe traumatic brain injury; severe cognitive impairment, disturbance of consciousness, or inability to complete the clinical scales or MRI examination; severe aphasia or cognitive impairment that prevented completion of psychiatric depression assessment; contraindications to MRI, including cardiac pacemaker, metallic implants, or claustrophobia; or a confirmed diagnosis of major psychiatric disorders before stroke, such as depressive disorder, bipolar disorder, or schizophrenia spectrum disorder.

### 2.3 Healthy control group: inclusion and exclusion criteria

#### 2.3.1 Inclusion criteria

Participants were eligible for the HC group if they met the following criteria: age 18–75 years; no history of stroke, transient ischemic attack, or other neurological disorders; no history of psychiatric disorders; right-handedness; and no use of medications affecting nervous system function within the previous 3 months, such as hypnotic drugs.

#### 2.3.2 Exclusion criteria

Healthy controls were excluded if they had contraindications to MRI, including cardiac pacemaker, metallic implants, or claustrophobia, or if cranial MRI revealed obvious structural abnormalities, such as tumor, marked brain atrophy, or hydrocephalus.

### 2.4 Clinical data collection and scale assessment

For the PSD group, the following data were collected: demographic information, including age, sex, and years of education; vascular risk factors, including hypertension, diabetes, and smoking; time since stroke; and medication information, including antidepressants, sedative-hypnotics, and antiepileptic drugs. Depression severity was assessed using the HAMD-17. Scores of 0–7 indicated no obvious depression, 8–17 mild depression, 18–24 moderate depression, and ≥25 severe depression. For the HC group, corresponding demographic information and scale screening results were collected. Anxiety symptoms were assessed with HAMA; patients with HAMA ≥7 were classified as having comorbid anxiety symptoms.

### 2.5 MRI data acquisition

All participants underwent MRI scanning on the same Philips MR Ingenia Elition 3.0 T scanner with a 32-channel phased-array head coil. Conventional T2-weighted imaging (T2WI) was first performed to exclude obvious structural abnormalities. High-resolution three-dimensional T1-weighted imaging (3D-T1WI) and resting-state functional magnetic resonance imaging (rs-fMRI) were then acquired using a single-shot gradient-echo echo-planar imaging (EPI) sequence. During scanning, participants were placed in the supine position. Foam pads and sponge earplugs were used to stabilize the head and reduce noise. During the rs-fMRI scan, participants were instructed to keep their eyes closed, remain awake and relaxed, avoid specific thoughts, and try not to fall asleep.

The acquisition parameters for 3D-T1WI were as follows: repetition time (TR) = 8.14 ms, echo time (TE) = 3.17 ms, inversion time (TI) = 450 ms, flip angle = 12°, field of view (FOV) = 256 × 256 mm, matrix = 256 × 256, slice thickness = 1 mm, slice gap = 0, and number of slices = 188.

The acquisition parameters for rs-fMRI were as follows: TR = 2000 ms, TE = 30 ms, flip angle = 90°, FOV = 220 × 220 mm, matrix = 64 × 64, slice thickness = 3 mm, slice gap = 1 mm, number of slices = 40, and number of time points = 180.

### 2.6 rs-fMRI preprocessing

Preprocessing was performed using the Data Processing and Analysis for Brain Imaging (DPABI) toolbox in MATLAB 2016a. DPABI provides standardized procedures for rs-fMRI preprocessing, head motion control, and quality management, which improves comparability across studies (13).

The preprocessing pipeline included the following steps. First, structural and functional images from each participant were visually inspected, and scans with obvious artifacts, incomplete coverage, or severe distortion were excluded. Second, the first 10 volumes of the rs-fMRI data were discarded to allow magnetization to reach a steady state and to reduce initial adaptation effects. Third, slice-timing correction was performed according to the slice acquisition order to align the time series of each voxel to the same reference time point. Fourth, head motion correction was conducted, and participants with translation >2.0 mm or rotation >2.0° were excluded. Framewise displacement (FD) was then calculated, and mean FD was included as a covariate in group comparisons and machine learning feature engineering (14). Fifth, individual T1WI images were coregistered to the functional images. T1WI images were then segmented into gray matter, white matter, and cerebrospinal fluid, and the functional images were normalized to Montreal Neurological Institute (MNI) space. Sixth, spatial smoothing was applied to the normalized functional images using a Gaussian kernel with a full width at half maximum (FWHM) of 6 mm to improve the signal-to-noise ratio and satisfy the statistical assumptions of random field theory. Seventh, linear drift was removed, and temporal band-pass filtering (0.01–0.08 Hz) was performed to retain low-frequency components more closely related to spontaneous neural activity at rest. Eighth, nuisance regression was performed to remove white matter signal, cerebrospinal fluid signal, and Friston-24 head motion parameters, thereby suppressing physiological noise (15).

### 2.7 Calculation of functional metrics and their biological significance

In this study, four complementary rs-fMRI-derived indices of spontaneous brain function were extracted: ALFF, ReHo, DC, and FC. Regions of interest (ROIs) were defined based on the widely used Automated Anatomical Labeling (AAL) atlas with 116 brain regions. The mean value within each ROI was extracted to represent regional functional activity and to improve measurement stability. These four indices characterize brain function at four levels, namely local amplitude, local synchrony, whole-brain hubness, and interregional coupling. Together, they may provide a more systematic description of the multiscale network abnormalities involved in PSD.

#### 2.7.1 ALFF

ALFF measures the intensity of spontaneous low-frequency fluctuations in the blood oxygen level-dependent (BOLD) signal at each voxel. It is widely regarded as an approximate index of local spontaneous neural activity and is commonly used in resting-state phenotypic studies of psychiatric disorders and cerebrovascular diseases (16). Using unfiltered data, the time series of each voxel was transformed into the frequency domain to obtain the power spectrum. The mean ALFF value for each brain region was then calculated within the low-frequency range of 0.01–0.08 Hz based on the AAL atlas.

#### 2.7.2 ReHo

ReHo measures the temporal consistency between a voxel and its neighboring voxels. It reflects the degree of local neural synchrony and is often interpreted as an index of local information processing or local functional integration (17). Using preprocessed but unsmoothed data, the time series of each voxel and those of all voxels within its spatial neighborhood (27-voxel neighborhood) were extracted, and the consistency statistic of these time series was calculated to obtain the ReHo value for that voxel. The mean ReHo value within each ROI was then calculated.

#### 2.7.3 Degree centrality

DC is a centrality measure derived from graph theory. It characterizes the strength of significant functional connections between a given voxel and all other voxels in the brain, and is used to identify hubs of the whole-brain functional network and alterations in network integration capacity (18). Using preprocessed but unsmoothed data, a voxel-wise correlation matrix was first calculated between each voxel and all other brain voxels. To suppress noisy connections, only correlations with r > 0.25 were retained. The sum of these correlation coefficients was defined as the DC value for each voxel. The mean DC value within each ROI was then calculated.

#### 2.7.4 Functional connectivity

FC reflects the temporal synchrony of spontaneous activity between different brain regions and is a core measure of brain network coupling and information communication (19). Pearson correlation coefficients were calculated between ROI pairs, followed by Fisher’s r-to-z transformation.

#### 2.7.5 Residualization of fMRI features

To minimize the potential influence of factors other than depressive symptoms on fMRI features, residualization was performed separately for each fMRI feature using a linear regression model. Age, sex, years of education, mean framewise displacement (mean FD), and the use of antidepressants, sedatives, and anxiolytics were included as covariates for all participants. Stroke-related covariates, including time since stroke, stroke type, lesion volume, lesion laterality, and primary lesion location, were included only in the residualization analysis of patients with stroke. The residualized fMRI features were then used in subsequent group comparisons and machine learning analyses:


$$X_{ij}\;=\;\alpha_{j\;}+\sum_{k=1}^{p}\beta_{kj}C_{ik}+\epsilon_{ij\;}$$

where *X_ij_* denotes the *j*th dynamic fMRI feature of the *i*th participant, *β_kj_* denotes the corresponding regression coefficient, *C_ik_* denotes the *k*th predefined confounding factor of the *i*th participant, and *ϵ_ij_* denotes the residualized dynamic fMRI feature after removal of the linear effects of confounding factors.

### 2.8 Machine learning: feature engineering, modeling, and interpretability

The objective of the machine learning analysis was to construct an interpretable classification model based on the differential functional phenotypes between PSD and HC, including ALFF, ReHo, DC, and FC features, in order to identify the key brain functional characteristics of PSD and to explain model decisions using SHapley Additive exPlanations (SHAP).

#### 2.8.1 Feature construction and preprocessing

The dataset was divided into training and test sets at a ratio of 7:3. Stratified sampling was used to preserve the proportion of PSD and HC participants in each set. Feature selection was performed exclusively within the training set. First, each fMRI feature was residualized according to clinical variables, head-motion parameters, and other predefined covariates, and Z scores were then calculated for all features. Two-sample t tests were subsequently used to identify fMRI features showing preliminary between-group differences. LASSO regression was then applied within the training set for further feature compression and selection. The penalty parameter was determined by cross-validation, and features with non-zero coefficients were retained for subsequent machine learning models (20).

#### 2.8.2 Model construction and SHAP interpretation

After standardization and feature selection, nine machine learning models were fitted on the training set: artificial neural network (ANN), support vector machine (SVM), k-nearest neighbors (KNN), Decision Tree, Light Gradient Boosting Machine (LightGBM), Gradient Boosting, Extra Trees, Random Forest, and Extreme Gradient Boosting (XGBoost). To ensure a fair comparison across algorithms, all models used the same training/test split, the same feature selection strategy in the training set, and the same performance evaluation metrics.

Within the training set, five-fold cross-validation was performed for hyperparameter tuning, with the area under the receiver operating characteristic curve (ROC-AUC) as the primary optimization metric. In each fold, the following procedure was repeated: standardization within the training fold, LASSO-based feature selection, model fitting, and prediction in the validation fold. This design ensured that both standardization and feature selection were nested within the cross-validation procedure and prevented data leakage. After hyperparameter optimization, each model was retrained on the full training set using the optimal parameters, and generalization performance was evaluated on the independent test set. The receiver operating characteristic (ROC) curve and AUC were treated as the primary outcomes. Accuracy, sensitivity, specificity, positive predictive value, negative predictive value, and F1 score were treated as secondary outcomes. The model with the highest test-set AUC was selected as the optimal model. SHAP was then applied to this model, and the features with the highest SHAP contributions were mapped back to specific brain regions and brain networks for neurobiological interpretation.

### 2.9 Statistical analysis

Continuous demographic variables were compared using the independent-samples t test or the Mann–Whitney U test, depending on data distribution. Categorical variables were compared using the χ² test or Fisher’s exact test, as appropriate (38). Between-group comparisons of ALFF, ReHo, DC, and FC features were performed using independent-samples t tests. To reduce the risk of false-positive findings, statistical significance for imaging-feature analyses was defined as a two-sided P < 0.001 (40).

## 3 Results

A total of 50 patients with PSD and 50 HC were included in this study. All participants completed rs-fMRI and were included in the subsequent imaging analyses and machine learning modeling. There were no significant differences in age, sex, or other general demographic characteristics between the two groups (P > 0.05; **Table 1**).

**Table 1:** Summary of demographic characteristics and clinical information of the participants.

Characteristics	PSD group (n = 50)	HC group (n = 50)	T / χ²	P
Age, years	64.04 ± 6.16	63.02 ± 5.75	0.856	0.394
Sex, male:female	27:23	26:24	0.174	0.677
Educational level, high school or below: above high school	31:19	33:17	0.4	0.841
Stroke type, Ischemic: Hemorrhagic	43:7	–	–	–
Lesion side, left:right	28:22	–	–	–
Disease duration, months	3.67 ± 0.71	–	–	–
HAMA score	17.66 ± 2.44	–	–	–
Depression severity, mild:moderate	29:21	–	–	–
Comorbid anxiety, n (%)	19 (38 %)	–	–	–

### 3.1 Multi-level resting-state functional abnormalities in patients with PSD

**Figure 1:**
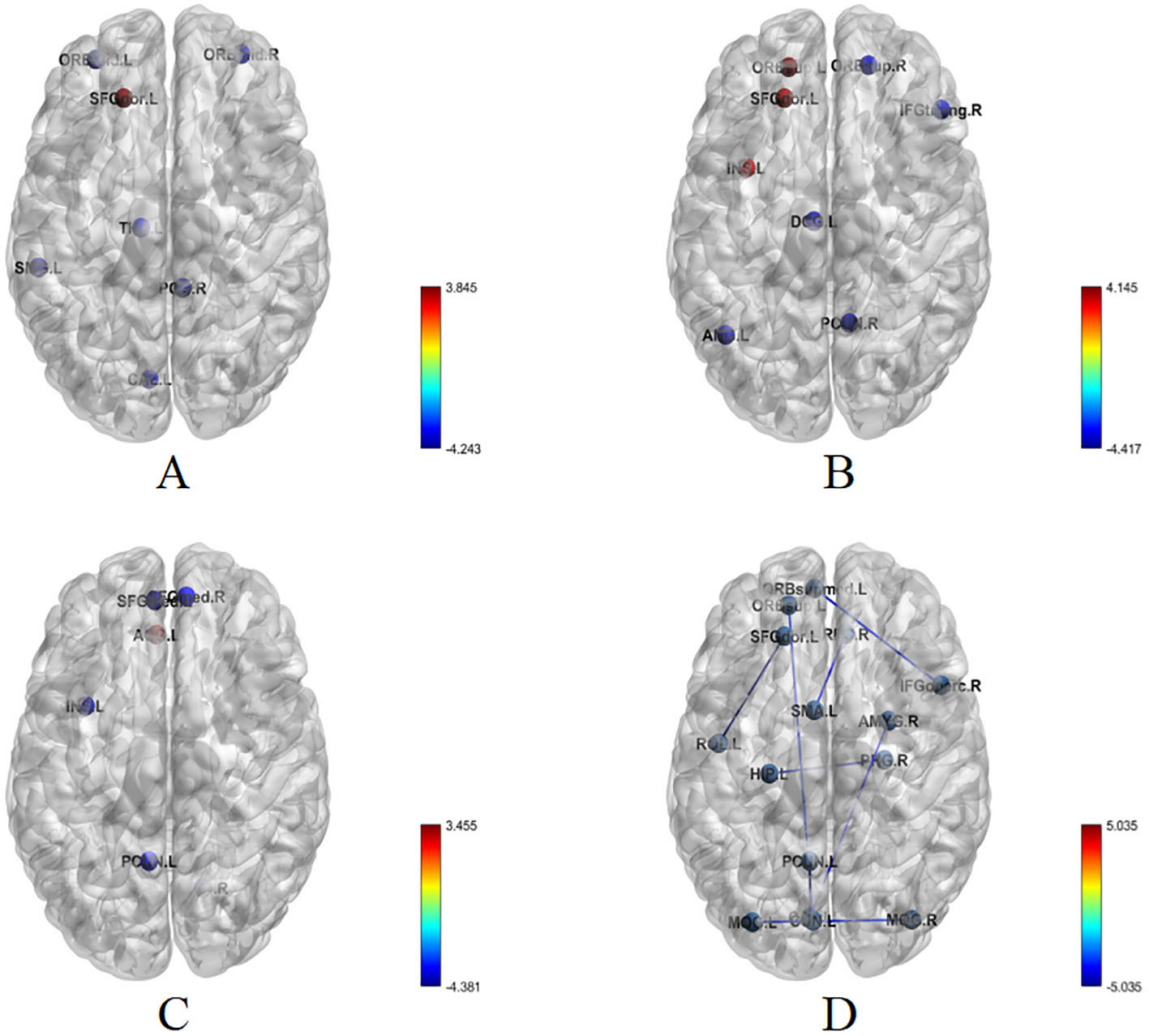
Multi-level resting-state functional abnormalities in patients with post-stroke depression. Brain maps showing significant differences in regional and inter-regional resting-state fMRI features between patients with post-stroke depression (PSD) and healthy controls (HC). Panels show group differences in **(A)** regional homogeneity (ReHo), **(B)** amplitude of low-frequency fluctuation (ALFF), **(C)** degree centrality (DC), and **(D)** ROI-to-ROI functional connectivity (FC). Warm colors indicate higher values in the PSD group relative to HC, whereas cool colors indicate lower values in the PSD group. In panel **(D)**, the connecting lines represent significantly altered functional connections, all of which showed reduced connectivity in PSD. Color bars denote t values. Statistical significance was defined as P < 0.001.

After controlling for demographic variables, head motion, and other potential confounders, regional functional indices and ROI-to-ROI functional connectivity analyses based on the AAL atlas identified 29 candidate imaging features with significant between-group differences. These included 7 ReHo features, 8 ALFF features, 6 DC features, and 8 functional connectivity features (**Figure 1**; **Table 2**).

**Table 2:** Group differences in regional fMRI metrics and ROI-to-ROI functional connectivity between PSD patients and healthy controls.

Metric	AAL abbreviation / connection	AAL anatomical label	MNI coordinate(s), x/y/z	T value	P value	Direction in PSD vs HC
ReHo	CAL.L	Left calcarine fissure and surrounding cortex	−7.14 / −78.67 / 6.44	−3.962	<0.001	Decreased
ReHo	THA.L	Left thalamus	−10.85 / −17.56 / 7.98	−3.508	<0.001	Decreased
ReHo	SFGdor.L	Left superior frontal gyrus, dorsolateral	−18.45 / 34.81 / 42.20	3.845	<0.001	Increased
ReHo	SMG.L	Left supramarginal gyrus	−55.79 / −33.64 / 30.45	−4.127	<0.001	Decreased
ReHo	PCG.R	Right posterior cingulate gyrus	7.44 / −41.81 / 21.87	−4.243	<0.001	Decreased
ReHo	ORBmid.L	Left middle frontal gyrus, orbital part	−30.65 / 50.43 / −9.62	−3.882	<0.001	Decreased
ReHo	ORBmid.R	Right middle frontal gyrus, orbital part	33.18 / 52.59 / −10.73	−3.607	<0.001	Decreased
ALFF	ANG.L	Left angular gyrus	−44.14 / −60.82 / 35.59	−3.835	<0.001	Decreased
ALFF	PCUN.R	Right precuneus	9.98 / −56.05 / 43.77	−4.417	<0.001	Decreased
ALFF	INS.L	Left insula	−35.13 / 6.65 / 3.44	3.513	<0.001	Increased
ALFF	ORBsup.L	Left superior frontal gyrus, orbital part	−16.56 / 47.32 / −13.31	4.145	<0.001	Increased
ALFF	SFGdor.L	Left superior frontal gyrus, dorsolateral	−18.45 / 34.81 / 42.20	3.822	<0.001	Increased
ALFF	ORBsup.R	Right superior frontal gyrus, orbital part	18.49 / 48.10 / −14.02	−4.025	<0.001	Decreased
ALFF	IFGtriang.R	Right inferior frontal gyrus, triangular part	50.33 / 30.16 / 14.17	−3.984	<0.001	Decreased
ALFF	DCG.L	Left median cingulate and paracingulate gyri	−5.48 / −14.92 / 41.57	−4.316	<0.001	Decreased
DC	PCUN.L	Left precuneus	−7.24 / −56.07 / 48.01	−3.497	<0.001	Decreased
DC	SFGmed.L	Left superior frontal gyrus, medial	−4.80 / 49.17 / 30.89	−4.381	<0.001	Decreased
DC	SFGmed.R	Right superior frontal gyrus, medial	9.10 / 50.84 / 30.22	−3.694	<0.001	Decreased
DC	ACG.L	Left anterior cingulate and paracingulate gyri	−4.04 / 35.40 / 13.95	3.455	<0.001	Increased
DC	INS.L	Left insula	−35.13 / 6.65 / 3.44	−3.953	<0.001	Decreased
DC	LING.R	Right lingual gyrus	16.29 / −66.93 / −3.87	−4.085	<0.001	Decreased
FC	PCUN.L – CAL.L	Left precuneus – left calcarine fissure and surrounding cortex	ROI1: −7.24 / −56.07 / 48.01; ROI2: −7.14 / −78.67 / 6.44	−5.035	<0.001	Decreased
FC	SFGdor.L – ROL.L	Left superior frontal gyrus, dorsolateral – left Rolandic operculum	ROI1: −18.45 / 34.81 / 42.20; ROI2: −47.16 / −8.48 / 13.95	−4.852	<0.001	Decreased
FC	IFGoperc.R – ORBsupmed.L	Right inferior frontal gyrus, opercular part – left superior frontal gyrus, medial orbital	ROI1: 50.20 / 14.98 / 21.41; ROI2: −5.17 / 54.06 / −7.40	−3.775	<0.001	Decreased
FC	PHG.R – HIP.L	Right parahippocampal gyrus – left hippocampus	ROI1: 25.38 / −15.15 / −20.47; ROI2: −25.03 / −20.74 / −10.13	−4.723	<0.001	Decreased
FC	AMYG.R – CAL.L	Right amygdala – left calcarine fissure and surrounding cortex	ROI1: 27.32 / 0.64 / −17.50; ROI2: −7.14 / −78.67 / 6.44	−3.958	<0.001	Decreased
FC	CUN.L – ORBsup.L	Left cuneus – left superior frontal gyrus, orbital part	ROI1: −5.93 / −80.13 / 27.22; ROI2: −16.56 / 47.32 / −13.31	−3.556	<0.001	Decreased
FC	SMA.L – REC.R	Left supplementary motor area – right gyrus rectus	ROI1: −5.32 / 4.85 / 61.38; ROI2: 8.35 / 35.64 / −18.04	−3.962	<0.001	Decreased
FC	MOG.L – MOG.R	Left middle occipital gyrus – right middle occipital gyrus	ROI1: −32.39 / −80.73 / 16.11; ROI2: 37.39 / −79.70 / 19.42	−3.673	<0.001	Decreased

AAL, automated anatomical labeling; ALFF, amplitude of low-frequency fluctuation; DC, degree centrality; FC, functional connectivity; HC, healthy control; MNI, Montreal Neurological Institute; PSD, post-stroke depression; ReHo, regional homogeneity.

ReHo analysis showed that, compared with the HC group, patients with PSD had significantly lower regional homogeneity in the left calcarine fissure and surrounding cortex, left thalamus, left supramarginal gyrus, right posterior cingulate gyrus, and bilateral orbital middle frontal gyri. In contrast, ReHo in the left dorsolateral superior frontal gyrus was significantly higher in the PSD group (P < 0.001; **Figure 1A**; **Table 2**).

ALFF analysis showed that patients with PSD had significantly lower low-frequency fluctuation amplitude in the left angular gyrus, right precuneus, right orbital superior frontal gyrus, right triangular part of the inferior frontal gyrus, and left median cingulate and paracingulate gyri. Higher ALFF was observed in the left insula, left orbital superior frontal gyrus, and left dorsolateral superior frontal gyrus (P < 0.001; **Figure 1B**; **Table 2**).

DC analysis further showed that patients with PSD had significantly lower degree centrality in the left precuneus, bilateral medial superior frontal gyri, left insula, and right lingual gyrus. By contrast, DC in the left anterior cingulate and paracingulate gyri was significantly higher in the PSD group (P < 0.001; **Figure 1C**; **Table 2**).

ROI-to-ROI functional connectivity analysis identified 8 inter-regional connections that were significantly lower in patients with PSD than in HCs. These connections were between the left precuneus and left calcarine fissure and surrounding cortex, left dorsolateral superior frontal gyrus and left Rolandic operculum, right opercular part of the inferior frontal gyrus and left medial orbital superior frontal gyrus, right parahippocampal gyrus and left hippocampus, right amygdala and left calcarine fissure and surrounding cortex, left cuneus and left orbital superior frontal gyrus, left supplementary motor area and right gyrus rectus, and bilateral middle occipital gyri (P < 0.001; **Figure 1D**; **Table 2**).

### 3.2 Stable discriminative features identified by LASSO regularization

**Figure 2:**
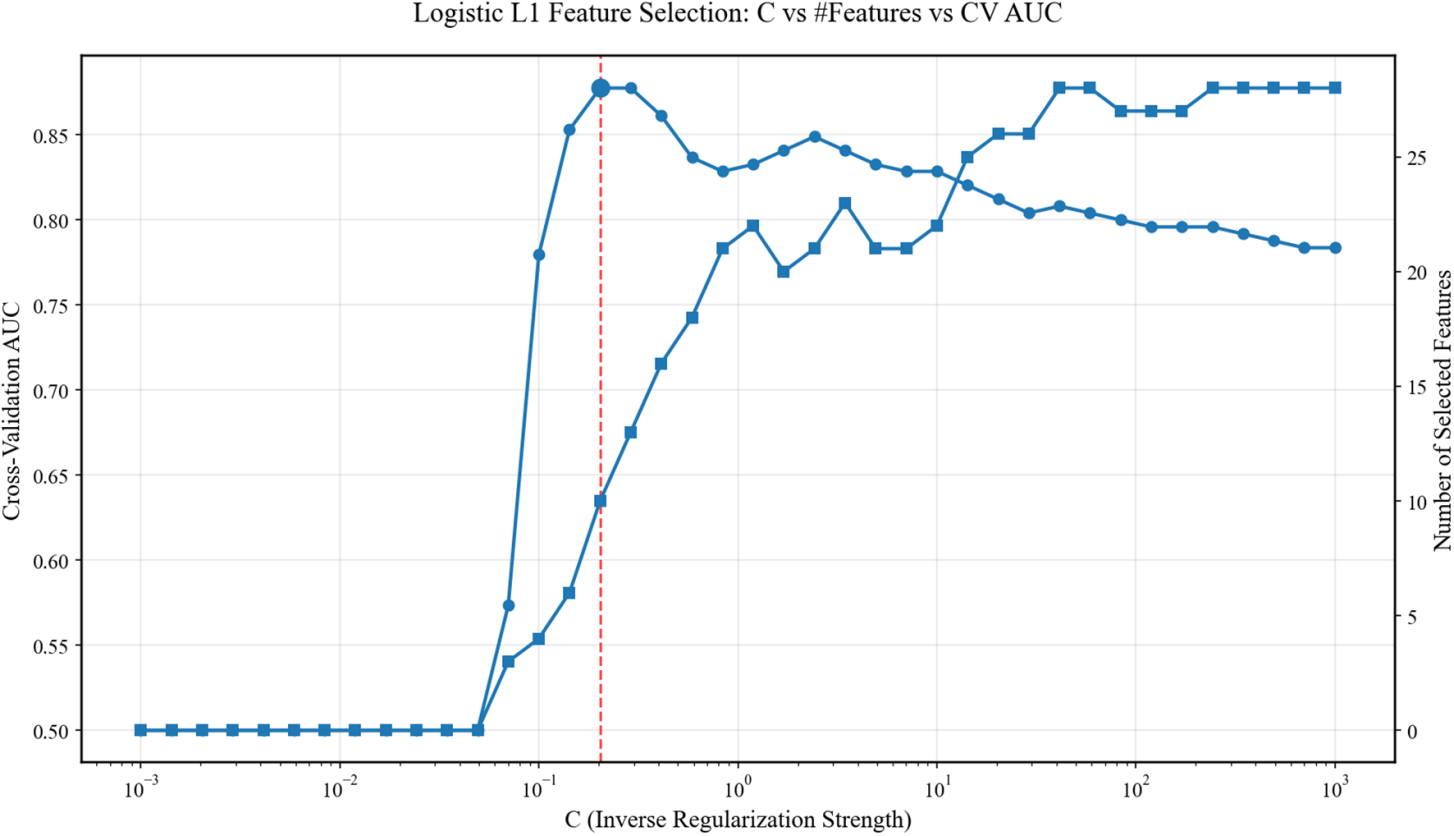
LASSO-based selection of discriminative rs-fMRI features for post-stroke depression. Least absolute shrinkage and selection operator (LASSO) logistic regression was applied to the candidate rs-fMRI features to reduce feature redundancy and multicollinearity. Cross-validated ROC-AUC values are plotted across different inverse regularization strengths (C), together with the corresponding number of retained features. The dashed vertical line indicates the optimal regularization parameter (C = 0.203), at which 10 non-zero features were retained and the cross-validated AUC reached 0.878.

To reduce feature redundancy and multicollinearity, an L1-regularized logistic regression model was applied to the 29 initially selected candidate features. Cross-validation showed that the optimal feature subset was obtained at a regularization parameter of C = 0.203. At this value, 10 features with non-zero coefficients were retained, and the cross-validated AUC was 0.878 (**Figure 2**). These findings indicate that a lower-dimensional set of core imaging features retained substantial discriminative capacity for PSD compared with the full set of candidate variables.

The 10 features retained for subsequent modeling were DC in the left anterior cingulate and paracingulate gyri (DC: ACG.L), ReHo in the left thalamus (ReHo: THA.L), ALFF in the left angular gyrus (ALFF: ANG.L), functional connectivity between the left precuneus and left calcarine fissure and surrounding cortex (FC: PCUN.L–CAL.L), ReHo in the left calcarine fissure and surrounding cortex (ReHo: CAL.L), ALFF in the left insula (ALFF: INS.L), ALFF in the right precuneus (ALFF: PCUN.R), DC in the right medial superior frontal gyrus (DC: SFGmed.R), ALFF in the left orbital superior frontal gyrus (ALFF: ORBsup.L), and ReHo in the left dorsolateral superior frontal gyrus (ReHo: SFGdor.L).

**Figure 3:**
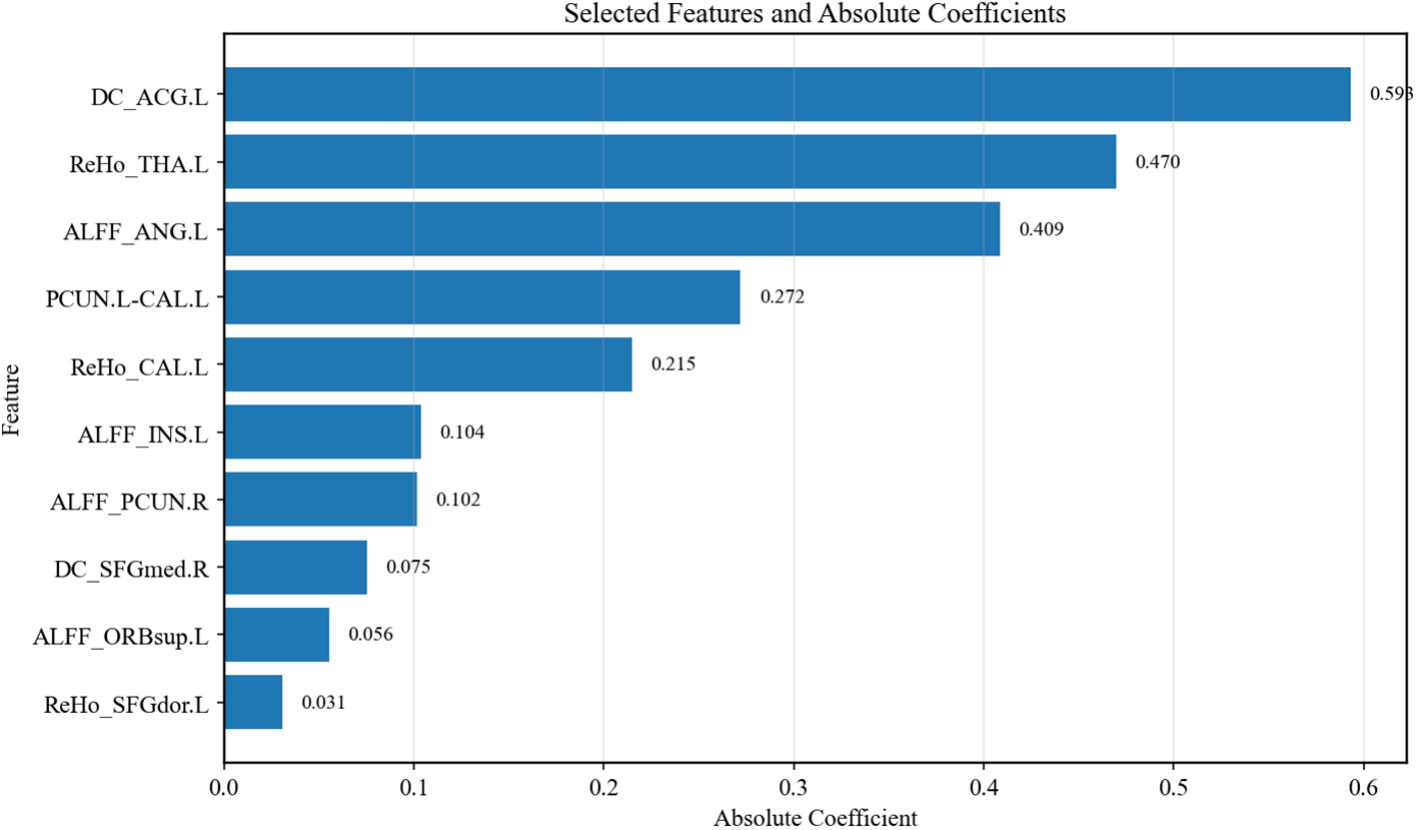
Relative importance of the final 10 LASSO-selected rs-fMRI features. The bar plot shows the absolute coefficients of the 10 features retained by LASSO regression. Higher absolute coefficients indicate greater contribution to discrimination between patients with post-stroke depression and healthy controls in the penalized logistic model. The most influential features were degree centrality in the left anterior cingulate and paracingulate gyri (DC : ACG.L), regional homogeneity in the left thalamus (ReHo : THA.L), and amplitude of low-frequency fluctuation in the left angular gyrus (ALFF : ANG.L), followed by functional connectivity between the left precuneus and left calcarine cortex (FC : PCUN.L–CAL.L).

Ranking based on the absolute LASSO coefficients showed that DC: ACG.L, ReHo: THA.L, and ALFF: ANG.L were the three highest-weighted features. These were followed by FC: PCUN.L–CAL.L and ReHo: CAL.L (**Figure 3**). In terms of feature composition, the final feature set included 4 ALFF features, 3 ReHo features, 2 DC features, and 1 FC feature. These features covered four functional levels: local spontaneous activity amplitude, local synchronization, network hub properties, and inter-regional coupling.

### 3.3 Performance comparison of machine learning models based on core imaging features

Using the 10 core imaging features, nine machine learning classifiers were constructed and compared. These included artificial neural network, support vector machine, K-nearest neighbors, decision tree, LightGBM, gradient boosting, Extra Trees (ET), random forest, and XGBoost. All models used the same training and test split, feature-selection procedure, and performance metrics to ensure comparability across classifiers.

**Figure 4:**
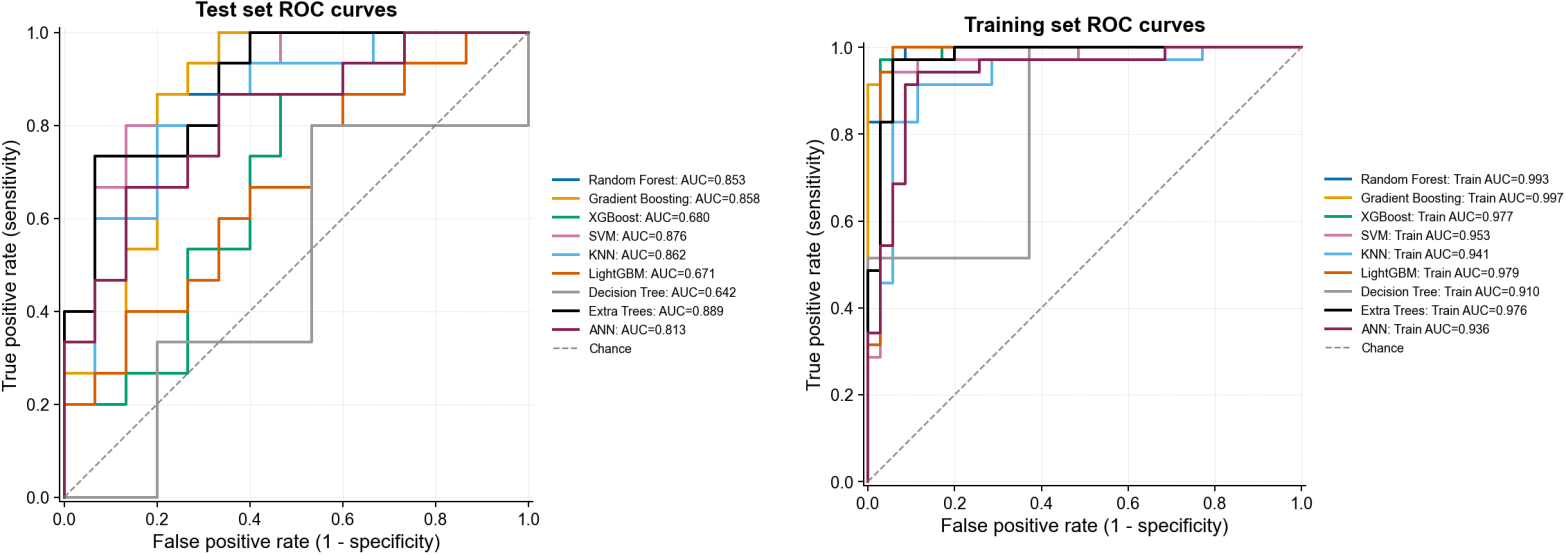
Receiver operating characteristic curves of machine-learning classifiers for identifying post-stroke depression. ROC curves are shown for the nine candidate classifiers trained using the same LASSO-selected rs-fMRI feature set. Model performance was evaluated in both the training and independent test sets. The diagonal line denotes chance-level discrimination. Among all models, the Extra Trees classifier achieved the highest ROC-AUC in the independent test set (AUC = 0.889), indicating the best overall discriminative performance according to the prespecified primary metric.

**Figure 5:**
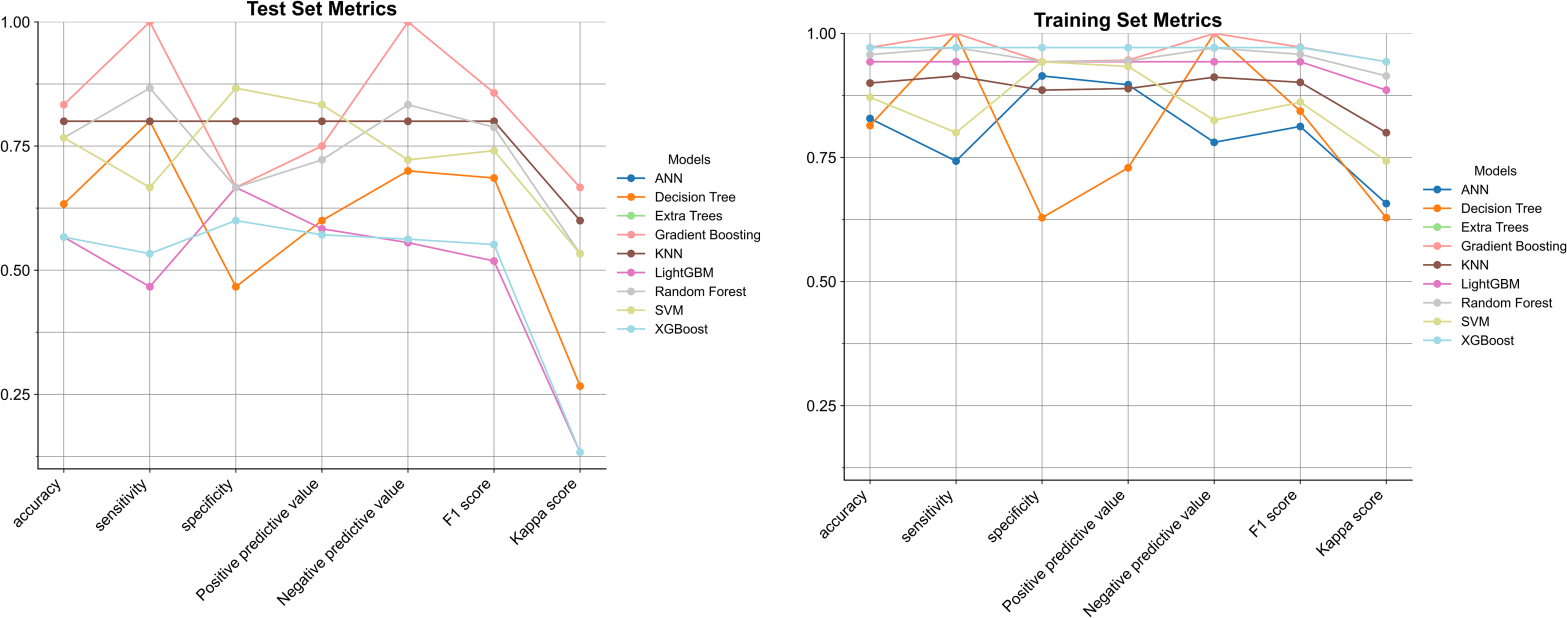
Comparative performance of the nine machine-learning classifiers. The radar plots summarize the classification performance of each model in the training and independent test sets. Metrics included ROC-AUC, accuracy, sensitivity, specificity, positive predictive value, negative predictive value, F1 score, and Cohen’s kappa. All models were evaluated using the same data split, feature-selection procedure, and performance metrics. Ensemble tree-based models, particularly Extra Trees and Random Forest, showed relatively stable performance across multiple indices, whereas Extra Trees yielded the highest test-set ROC-AUC.

In the independent test set, the ET model achieved the highest ROC-AUC, with a test-set AUC of 0.889 (**Figure 4**). It was therefore selected as the best-performing classifier in this study. In addition to AUC, model performance was evaluated using accuracy, sensitivity, specificity, positive predictive value, negative predictive value, F1 score, and kappa coefficient (**Figure 5**). Overall, ensemble tree-based models, particularly ET and random forest, performed relatively well across several metrics. According to the prespecified primary metric of ROC-AUC, however, ET showed the best discriminative performance in the independent test set.

### 3.4 SHAP-based interpretability analysis of the ET model

**Figure 6:**
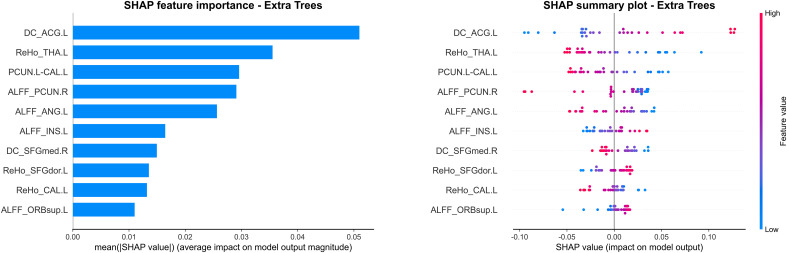
SHAP-based interpretation of the Extra Trees classifier. SHapley Additive exPlanations (SHAP) analysis was performed to interpret the decision structure of the best-performing Extra Trees model. The left panel shows global feature importance ranked by the mean absolute SHAP value, whereas the right panel shows the distribution of individual SHAP values for each feature. Positive SHAP values indicate contributions toward classification as post-stroke depression, and negative values indicate contributions away from this classification. The most influential features included DC: ACG.L, ReHo: THA.L, FC: PCUN.L–CAL.L, ALFF: PCUN.R, and ALFF: ANG.L, suggesting that the model decision was primarily driven by abnormalities involving the cingulo-thalamic circuit, posterior default-mode regions, and visual-associated functional connectivity.

To further characterize the decision structure of the optimal model, SHAP analysis was performed for the ET classifier. Global SHAP importance ranking showed that DC in the left anterior cingulate and paracingulate gyri (DC: ACG.L) contributed most to model output. This was followed by ReHo in the left thalamus (ReHo: THA.L), functional connectivity between the left precuneus and left calcarine fissure and surrounding cortex (FC: PCUN.L–CAL.L), ALFF in the right precuneus (ALFF: PCUN.R), and ALFF in the left angular gyrus (ALFF: ANG.L) (**Figure 6**). These features involved the cingulo-thalamo-frontal circuit, posterior nodes of the default mode network, visual-associated regions, and insula-related networks. This pattern suggests that the classification decisions of the ET model were neurobiologically traceable.

### 3.5 Brain functional differences according to depression severity in patients with PSD

To further explore functional alterations associated with depression severity, patients with PSD were divided into mild and moderate PSD groups according to HAMD-17 scores. Between-group comparisons were performed for ALFF, ReHo, DC, and ROI-to-ROI functional connectivity. At the prespecified statistical threshold of P < 0.001, significant group differences were observed in ALFF and functional connectivity. No ReHo or DC features reached this threshold.

**Figure 7:**
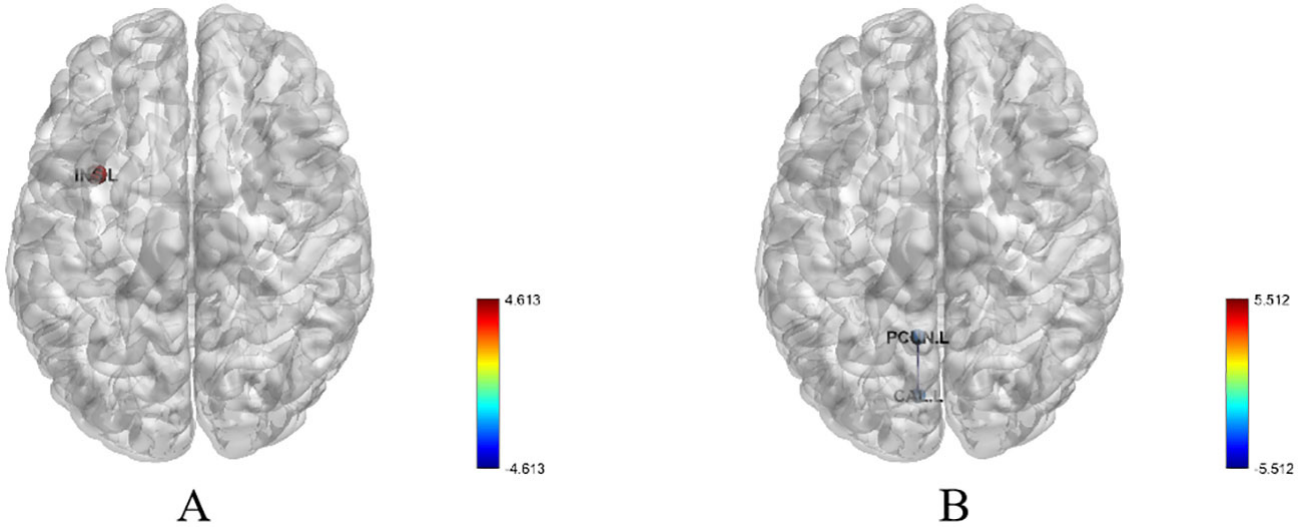
Severity-related alterations in resting-state brain function among patients with post-stroke depression. Brain maps showing functional differences between patients with moderate and mild post-stroke depression. **(A)** Compared with the mild PSD group, the moderate PSD group showed increased ALFF in the left insula. **(B)** The moderate PSD group showed reduced functional connectivity between the left precuneus and the left calcarine fissure and surrounding cortex. Color bars denote t values.

Compared with the mild PSD group, the moderate PSD group showed significantly higher ALFF in the left insula (INS.L; MNI coordinates: x = −35.13, y = 6.65, z = 3.44; t = 4.613, P < 0.001; **Figure 7A**). This finding indicates that greater depression severity may be accompanied by increased local spontaneous neural activity in the insula.

Functional connectivity analysis showed that the moderate PSD group had significantly lower connectivity between the left precuneus (PCUN.L; MNI coordinates: x = −7.24, y = −56.07, z = 48.01) and the left calcarine fissure and surrounding cortex (CAL.L; MNI coordinates: x = −7.14, y = −78.67, z = 6.44) compared with the mild PSD group (t = −5.512, P < 0.001; **Figure 7B**).

## 4 Discussion

In this study, multi-level resting-state fMRI measures and interpretable machine learning were used to examine functional brain differences between patients with PSD and healthy controls. The main findings indicate that patients with PSD showed distributed alterations involving the cingulate cortex, thalamus, prefrontal cortex, insula, angular gyrus, precuneus, and visual-associated regions, rather than changes confined to a single region or network. Ten core imaging features selected by LASSO differentiated patients with PSD from healthy controls with relatively good performance, and the Extra Trees model achieved the highest discriminative performance in the independent test set. SHAP analysis further indicated that model decisions were mainly driven by DC in the left anterior cingulate and paracingulate gyri, ReHo in the left thalamus, functional connectivity between the left precuneus and left calcarine cortex, ALFF in the right precuneus, and ALFF in the left angular gyrus. Given the absence of a post-stroke non-depressed control group, these findings should be interpreted as functional differences associated with PSD relative to healthy controls. They may reflect a combination of stroke-related network reorganization and depressive symptom-related functional alterations.

At the network level, the present findings are broadly consistent with the view that PSD is associated with distributed functional network abnormalities after stroke. Previous studies have reported altered intra-network and inter-network functional connectivity in patients with post-ischemic stroke depression, particularly involving limbic regions, the prefrontal cortex, and posterior nodes of the default mode network (21). Altered connectivity among the salience, default mode, and frontoparietal networks has also been observed in PSD, suggesting that depressive symptoms after stroke may be related to disturbed interactions among internal affective processing, external attentional allocation, and cognitive control (22). Building on these observations, the present study integrated ALFF, ReHo, DC, and FC measures. The results suggest that local spontaneous activity, local synchronization, whole-brain hub properties, and inter-regional coupling all contributed to the distinction between PSD and healthy controls. This pattern indicates that functional differences in PSD are unlikely to be limited to reduced connectivity in a single pathway. Instead, they may involve multi-level alterations across emotion regulation, cognitive control, and sensory integration systems.

DC in the left anterior cingulate and paracingulate gyri was the most important SHAP feature in this study. The anterior cingulate cortex is positioned at the interface of affective evaluation, conflict monitoring, motivational regulation, and autonomic responses. Higher degree centrality in this region may indicate a redistribution of its network connectivity weight in patients with PSD. This finding should not be interpreted simply as functional enhancement. A more cautious interpretation is that it may reflect altered hub-like involvement of the cingulate region within emotion-regulation circuits after stroke. Studies based on focal brain lesions have suggested that depressive symptoms are not always determined by damage to a fixed anatomical site, but may relate to lesions connected to a shared depression-associated circuit (23). Lesion-symptom studies in PSD have also shown that different depressive symptom dimensions are associated with distinct structural pathways and functional networks, indicating the limitations of explaining PSD solely by lesion location (24). In this context, the contribution of anterior cingulate DC in the present model supports a network-based interpretation of PSD-related differences, while remaining compatible with the possibility that these alterations partly reflect broader stroke-related network effects.

Reduced ReHo in the left thalamus was another high-weighted feature. The thalamus is an important relay structure for cortico-cortical and cortico-limbic communication, and is involved in arousal, emotional responses, attentional switching, and interoceptive integration. Lower local synchronization in the left thalamus may indicate altered regional temporal coherence, which could affect thalamic modulation of prefrontal, cingulate, and limbic activity. A recent study reported abnormal intrinsic functional hubs and seed-based connectivity in patients with PSD, involving the default mode network, limbic system, and cognitive control-related regions (25). This is consistent with the co-occurrence of increased anterior cingulate DC and reduced thalamic ReHo observed in the present study. Together, these findings suggest that patients with PSD may show a pattern of increased involvement of regulatory hub regions alongside reduced thalamic regional coherence. Such a pattern could be related to less efficient information transfer across emotion-related networks and greater demands on cognitive control systems during internal emotional processing.

The present study also observed functional alterations in posterior default mode and visual-associated regions. These included lower ALFF in the left angular gyrus, lower ALFF in the right precuneus, lower ReHo in the left calcarine fissure and surrounding cortex, and reduced functional connectivity between the left precuneus and left calcarine cortex. The angular gyrus and precuneus are important posterior nodes of the default mode network and are involved in self-referential processing, episodic memory, attentional shifting, and integration of internal and external information. A large-scale study of recurrent major depressive disorder reported reduced functional connectivity within the default mode network, suggesting that altered posterior default mode function may be relevant to self-referential processing and emotion regulation (26). A multicenter mega-analysis also supported the presence of distributed functional connectivity abnormalities in major depressive disorder, rather than abnormalities restricted to a single brain region (27). In the present study, lower activity in the angular gyrus and precuneus may indicate reduced baseline activity in posterior default mode regions in patients with PSD relative to healthy controls. This difference may be related to impaired integration of internal emotional experience and external environmental information.

Reduced connectivity between the precuneus and calcarine cortex is a noteworthy finding. The calcarine fissure and surrounding cortex are usually regarded as primary visual processing regions. However, visual cortices also participate in emotional visual processing, attentional allocation, and visual memory retrieval through interactions with default mode and limbic systems. The reduced connectivity between the left precuneus and left calcarine cortex observed in patients with PSD may suggest altered coupling between visual-associated processing and self-referential networks. Previous fALFF research has reported frequency-specific alterations in local spontaneous activity in PSD, with left insular fALFF associated with depression severity (28). More recent work also showed frequency-dependent and time-varying abnormalities of low-frequency activity in PSD, indicating that local spontaneous activity may capture dynamic aspects of post-stroke depressive symptoms (29). By considering visual cortex ReHo, precuneus ALFF, and precuneus-calcarine FC within the same framework, the present study suggests that sensory-default mode coupling may be a relevant but underexamined feature in PSD-related functional alterations.

The insula-related findings provide additional information about depression severity within the PSD group. In the present study, ALFF in the left insula was higher in patients with PSD than in healthy controls, and was further increased in patients with moderate PSD compared with those with mild PSD. The insula is a core node of the salience network and is involved in interoception, somatic experience, emotional salience evaluation, and autonomic responses. Previous resting-state fMRI research reported altered functional connectivity between the anterior insula and the superior frontal gyrus, middle frontal gyrus, and orbitofrontal cortex in patients with PSD, with associations with depression severity (30). fALFF findings have also suggested that higher post-stroke depression scores are associated with greater spontaneous activity in the left insula (28). The present results are consistent with these observations and suggest that increased insular activity may be related to sustained processing of internal bodily signals and negative emotional salience in PSD. At the same time, the present study also found reduced DC in the left insula. This may indicate a dissociation between increased local spontaneous activity and reduced whole-brain network integration. In the context of post-stroke brain network reorganization, increased local reactivity does not necessarily imply more efficient network-level regulation.

Alterations in prefrontal and orbitofrontal regions further suggest that PSD is associated with differences in brain systems involved in emotion regulation and reward processing. In this study, several indices related to the orbital superior frontal gyrus, dorsolateral superior frontal gyrus, medial superior frontal gyrus, and inferior frontal gyrus showed between-group differences, and some of these features were retained in the final machine learning model. The dorsolateral prefrontal cortex is involved in cognitive control and emotion reappraisal. The orbitofrontal cortex contributes to reward valuation and negative feedback processing, whereas the medial prefrontal cortex is closely related to self-referential emotional processing. Altered interactions between reward networks and the default mode network have been reported in PSD, particularly involving abnormal coupling between nucleus accumbens-related networks and the default mode network, which may be relevant to anhedonia and reduced motivation (31). Therefore, the co-occurring alterations in frontal, insular, cingulate, and posterior default mode regions in the present study may reflect differences in the coordination of emotional salience, reward processing, and cognitive control in patients with PSD compared with healthy controls.

The value of the machine learning analysis in this study lies not only in classification performance, but also in the ability to map discriminative features back to interpretable neural systems. LASSO was used to reduce feature dimensionality, and nine models were compared under the same analytic framework. The ET model was selected as the optimal model according to the prespecified primary metric. Importantly, SHAP analysis indicated that model discrimination mainly depended on brain regions and connections with plausible neurobiological relevance, rather than on a large number of difficult-to-interpret high-dimensional features. Neuroimaging biomarker research has emphasized that classification models are most useful when they are reproducible, interpretable, and have a plausible path toward clinical translation (32). Studies of machine learning using resting-state fMRI have also noted that feature construction, cross-validation, sample size, and external validation substantially affect model generalizability (33). In the present study, within-training-set feature selection, regularized dimensionality reduction, and independent test-set evaluation were used to reduce the risk of overfitting. Nevertheless, the main contribution of the model should be viewed as an exploratory identification of PSD-associated functional imaging features, rather than as a diagnostic tool ready for clinical application.

The clinical relevance of this study is that it provides multi-level functional imaging evidence of brain differences associated with PSD relative to healthy controls. Previous neuroimaging prediction studies have shown that individual-level models are vulnerable to sample heterogeneity, feature leakage, and insufficient validation (34). Recent discussions of explainable artificial intelligence in psychiatry have also emphasized that model outputs need to be understandable to both clinicians and mechanism-oriented researchers before they can support translational work (35). In the present study, the highest-ranking SHAP features were concentrated in the cingulo-thalamo-prefrontal/insular circuit and posterior default mode-visual pathway. These features are broadly consistent with clinical manifestations of PSD, although they should not be regarded as sufficient for standalone diagnosis. PSD has been consistently associated with poorer functional outcomes, and a recent systematic review also supported an association between PSD and post-stroke recovery (36). Therefore, early identification of functional brain patterns associated with depressive symptoms after stroke may help refine screening strategies and inform the timing of post-stroke emotional assessment and intervention.

Future PSD research should move beyond static group comparisons and incorporate longitudinal, dynamic, and multimodal approaches. Recent multimodal MRI research has shown that structural measures, white matter microstructure, and spontaneous brain activity may jointly characterize PSD-related alterations, suggesting that a single imaging index is unlikely to capture the full neurobiological context of PSD (37, 38). Another longitudinal study suggested that impaired neurovascular coupling and reduced glymphatic system function after stroke may contribute to depressive symptoms, providing a more physiologically grounded framework for interpreting BOLD signal changes (39, , 40). The ALFF, ReHo, DC, and FC alterations observed in the present study may provide candidate regions for future studies integrating perfusion imaging, diffusion MRI, lesion network mapping, and inflammatory markers. In particular, the anterior cingulate, thalamus, insula, angular gyrus, precuneus, and calcarine-associated pathways warrant further evaluation in larger samples and longitudinal cohorts to determine their relevance to PSD onset, symptom progression, and treatment response.

This study has several limitations. First, it was a single-center case-control study with a relatively limited sample size. Although within-training-set feature selection, regularized dimensionality reduction, and independent test-set evaluation were used, the machine learning models may still be influenced by sample composition and center-specific effects. Future studies require external validation in larger multicenter cohorts. Model stability should also be assessed across different scanners, stroke subtypes, and post-stroke stages. Second, the present study primarily compared patients with PSD and healthy controls, without including a parallel group of post-stroke patients without depression. Therefore, some of the observed functional differences may reflect the effects of stroke itself, depressive symptoms, or both. Although lesion volume, lesion laterality, time since stroke, medication use, and other factors were included in residualization where appropriate, residual confounding cannot be fully excluded. Future studies should include healthy controls, post-stroke non-depressed patients, and patients with PSD to better distinguish stroke-related alterations from depression-related alterations. Third, ROI-level features were extracted using the AAL atlas. This approach improves feature stability and interpretability, but may reduce sensitivity to functional subdivisions within specific regions. The anterior cingulate cortex, insula, thalamus, precuneus, and visual cortex all contain functionally heterogeneous subregions. Future work may benefit from finer parcellation schemes, individualized network mapping, and dynamic functional connectivity analyses to improve spatial and temporal characterization of PSD-related functional phenotypes. Fourth, this study was based primarily on cross-sectional resting-state fMRI data. Therefore, causal relationships between functional brain differences and depressive symptoms after stroke cannot be inferred.

## 5 Conclusion

This study showed that patients with PSD differed from healthy controls across multiple levels of resting-state functional organization, particularly involving the cingulo-thalamo-prefrontal/insular circuit and posterior default mode-visual network. Core imaging features identified through interpretable machine learning showed plausible neurobiological relevance and may serve as candidate markers for future studies of PSD-related brain function and individualized risk stratification. Further multicenter longitudinal studies including post-stroke non-depressed controls and multimodal imaging are needed to clarify the specificity, temporal evolution, and clinical utility of these features in PSD.

## Data Availability

The raw data supporting the conclusions of this article will be made available by the authors, without undue reservation.
